# Pharyngeal pouches provide a niche microenvironment for arch artery progenitor specification

**DOI:** 10.1242/dev.192658

**Published:** 2021-01-20

**Authors:** Aihua Mao, Mingming Zhang, Linwei Li, Jie Liu, Guozhu Ning, Yu Cao, Qiang Wang

**Affiliations:** 1 State Key Laboratory of Membrane Biology, Institute of Zoology, University of Chinese Academy of Sciences, Chinese Academy of Sciences, Beijing 100101, China; 2 Institute for Stem Cell and Regeneration, Chinese Academy of Sciences, Beijing 100101, China

**Keywords:** Pharyngeal arch artery morphogenesis, Progenitor specification, Pharyngeal pouch, BMP signal, Niche microenvironment

## Abstract

The paired pharyngeal arch arteries (PAAs) are transient blood vessels connecting the heart with the dorsal aorta during embryogenesis. Although PAA malformations often occur along with pharyngeal pouch defects, the functional interaction between these adjacent tissues remains largely unclear. Here, we report that pharyngeal pouches are essential for PAA progenitor specification in zebrafish embryos. We reveal that the segmentation of pharyngeal pouches coincides spatiotemporally with the emergence of PAA progenitor clusters. These pouches physically associate with pharyngeal mesoderm in discrete regions and provide a niche microenvironment for PAA progenitor commitment by expressing BMP proteins. Specifically, pouch-derived BMP2a and BMP5 are the primary niche cues responsible for activating the BMP/Smad pathway in pharyngeal mesoderm, thereby promoting progenitor specification. In addition, BMP2a and BMP5 play an inductive function in the expression of the *cloche* gene *npas4l* in PAA progenitors. *cloche* mutants exhibit a striking failure to specify PAA progenitors and display ectopic expression of head muscle markers in the pharyngeal mesoderm. Therefore, our results support a crucial role for pharyngeal pouches in establishing a progenitor niche for PAA morphogenesis via BMP2a/5 expression.

## Abstract (precis)

**Summary:** Pharyngeal pouch-derived BMP2a and BMP5 are the key niche cues that induce arch artery progenitor specification during zebrafish embryonic development.

## INTRODUCTION

During vertebrate development, the pharyngeal arch arteries (PAAs), also known as aortic arch arteries, are transient embryonic blood vessels that connect the heart to the dorsal aorta (Hiruma et al., 2002). These arteries form in a cranial-to-caudal sequence, and in mammals (or amniotes) are followed by the regression of the first and second PAAs, whereas the PAA3, PAA4 and PAA6 undergo asymmetric remodeling and contribute to the carotid arteries and great vessels of the heart, including the aorta and pulmonary arteries (Congdon, 1922; Hiruma et al., 2002). Improper embryonic development of the PAAs may cause life-threating congenital cardiovascular defects that are frequently of unknown etiology (Abrial et al., 2017; Srivastava, 2001). The regulatory mechanisms involved in PAA remodeling have been studied extensively (Abu-Issa et al., 2002; Kameda, 2009; Liu et al., 2004; Papangeli and Scambler, 2013; Watanabe et al., 2010); however, the cellular events and genetic control of PAA formation are just beginning to be unveiled.

Embryos of the model vertebrate zebrafish exhibit similar processes of PAA morphogenesis, despite the absence of aortic arch remodeling (Anderson et al., 2008; Isogai et al., 2001). During zebrafish mid-somitogenesis, a common mesodermal progenitor population, which is segregated from the cardiac precursors in the heart field, can give rise to PAAs, head muscles (HM) and cardiac outflow tract (OFT) (Guner-Ataman et al., 2018). These common progenitors are housed in different pharyngeal arches and exhibit distinct gene expression profiles prior to the morphogenesis of PAAs, HM and cardiac OFT (Nagelberg et al., 2015; Paffett-Lugassy et al., 2017). In particular, the common progenitors located in PAAs 3-6 condensed into *nkx2.5^+^* clusters in a craniocaudal sequence and then gave rise to PAA endothelium, implying the progressive emergence of PAA progenitors (Paffett-Lugassy et al., 2013). Specifically, cell lineage-tracing experiments showed that progenitors for PAA5 and PAA6 were specified from the pharyngeal mesoderm after 30 hpf, a time point much later than that of the segregation of the common progenitors (Paffett-Lugassy et al., 2013). Thus, these common progenitors might undergo further specification in the pharyngeal region, a hypothesis that remains to be evaluated experimentally.

Interestingly, the homeodomain transcription factor *nkx2.5* is expressed in presumptive PAA endothelial progenitors; however, it is dispensable for PAA progenitor specification (Paffett-Lugassy et al., 2013). Subsequently, chemical inhibition experiments suggest that transforming growth factor β (TGFβ) signaling is required for the differentiation of PAA progenitors into angioblasts (Abrial et al., 2017). In addition, transcription factors *etv2* and *scl* have been shown to be essential for the initiation of the angioblast program (Sumanas and Lin, 2006). However, the molecular mechanism underlying PAA progenitor specification in the pharyngeal region has yet to be fully investigated.

Endodermal pouches are a series of outpocketings budding from the developing foregut (Graham and Smith, 2001). Interestingly, affected arch arteries often occur simultaneously with pouch defects, possibly because of their close physical relation and potential interactions during development (Li et al., 2012; Wendling et al., 2000). Because the pharyngeal pouches express several signaling molecules that participate in the patterning of the pharyngeal skeleton and in the specification of the arch-associated ganglia, their roles in aortic arch morphogenesis have been traditionally considered as secondary (Crump et al., 2004; Holzschuh et al., 2005; Ning et al., 2013). Intriguingly, our recent study indicated an indispensable role for PDGF signaling from pharyngeal pouches in the PAA angioblast proliferation (Mao et al., 2019), but whether pouch endoderm directly functions in PAA progenitor specification remains unknown. In this work, we further found that pouch-derived BMP signaling is necessary for the specification of PAA progenitors.

## RESULTS

### ZsYellow^+^ pharyngeal mesoderm contains distinct vascular progenitor subpopulations

PAAs originate from a fraction of *nkx2.5*^+^ cells within the heart field (Guner-Ataman et al., 2018; Nagelberg et al., 2015; Paffett-Lugassy et al., 2013). To meticulously observe cell behaviors during the formation of these PAAs, time-lapse confocal imaging studies were performed in *Tg(nkx2.5:ZsYellow)* embryos from 22 hpf. At this time point, some cells in the ZsYellow^+^ pharyngeal mesoderm started to pile up in the ventral root of the prospective third aortic arch, and then sprouted dorsally by 24 hpf (Fig. S1A). This process was repeated for PAA4-PAA6 in a cranial-to-caudal sequence from 28 to 42 hpf (Fig. S1A), which is consistent with previous observations (Paffett-Lugassy et al., 2013).

During somitogenesis, the common progenitors of PAAs, HM and cardiac OFT are specialized and remain lateral with consecutive *nkx2.5* expression when cardiac precursors migrate medially (Guner-Ataman et al., 2018; Paffett-Lugassy et al., 2013). Interestingly, the PAA progenitor clusters sequentially emerged at discrete positions in the pharyngeal mesoderm to form aortic arches, whereas the ZsYellow*^+^* cells between the PAA progenitor clusters seemed to preserve their locations and would not contribute to PAAs (Fig. S1A,B). These observations support that the pharyngeal mesoderm within pharyngeal arches 3-6 might be further specified into different subpopulations.

**Fig. 1.**
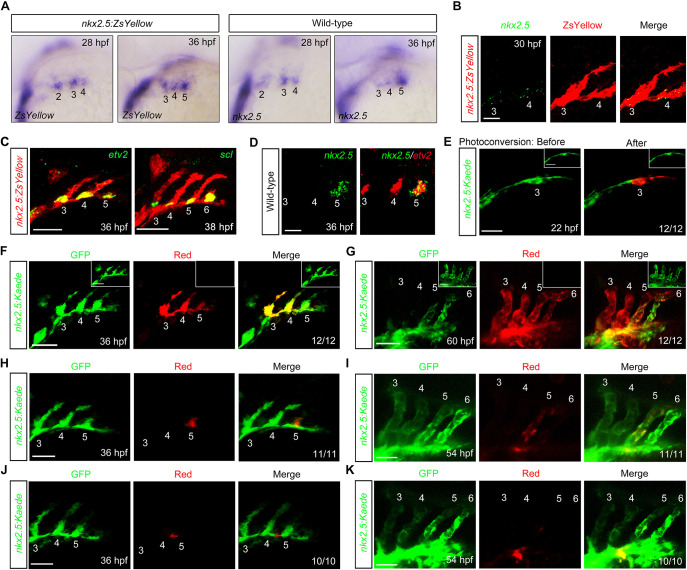
**Pharyngeal mesoderm contains two vascular progenitor subpopulations.** (A) *In situ* hybridization analysis of the expression of *ZsYellow* and *nkx2.5*. Numbers indicate PAA clusters. Lateral views with anterior on the left. (B,C) The expression patterns of the PAA progenitor marker *nkx2.5* (B), and angioblast markers *scl* and *etv2* (C) in *Tg(nkx2.5:ZsYellow)* embryos were detected by fluorescent *in situ* hybridization. Embryos were first subjected to fluorescent *in situ* hybridization and then to immunostaining with anti-ZsYellow antibody. Scale bar: 20 μm in B; 50 μm in C. (D) Colocalization analysis of *nkx2.5* and *etv2* by double fluorescent *in situ* hybridization. Scale bar: 20 μm. (E-G) The Kaede proteins in PAA cluster 3 and the subsequent posterior pharyngeal mesoderm on the right side of the *Tg(nkx2.5:kaede)* embryos were photoconverted at 22 hpf (E). Cells on the left side of the pharyngeal mesoderm remained unphotoconverted as an internal control (inset). Embryos were subsequently imaged in the green and red channels at 36 hpf (F) and 60 hpf (G). Scale bars: 50 μm. (H-K) Localized photoconversion of PAA cluster 5 (H) or Kaede^+^ cells between cluster 4 and 5 (J) at 36 hpf. Red, green and merged images of the same embryos at 54 hpf are shown in I and K. Scale bars: 50 μm. The photoconversion experiments in E-K were repeated three times independently, and three to five embryos per group were used each time. All the embryos analyzed showed similar distributions of photoconverted cells. The Kaede^+^ cells in PAA cluster 5 and between PAA clusters 4 and 5 gave rise to PAA5 and ventral aorta, respectively, indicating that the pharyngeal mesoderm contains two vascular progenitor subpopulations.

To test this hypothesis, we evaluated the expression pattern of *nkx2.5*, the specific marker of PAA progenitors from 28 hpf to 38 hpf (Guner-Ataman et al., 2018; Nagelberg et al., 2015; Paffett-Lugassy et al., 2013). As described in previous report (Paffett-Lugassy et al., 2013), *ZsYellow* transcripts derived from *nkx2.5* cis-regulatory sequences in *Tg(nkx2.5:ZsYellow)* embryos gradually appeared in the progenitor clusters (Fig. 1A). The endogenous *nkx2.5* transcripts were also sequentially observed, but eventually decreased when the PAA progenitors differentiated into angioblasts (Fig. 1A). Importantly, the transcripts of both *ZsYellow* and *nkx2.5* were enriched in the PAA progenitor clusters, showing a discontinuous distribution (Fig. 1A). We further combined immunofluorescence and fluorescence *in situ* hybridization experiments, and found that most of the *nkx2.5^+^* progenitors were restricted to the PAA clusters within ZsYellow^+^ pharyngeal mesoderm (Fig. 1B). The different expression patterns between endogenous *nkx2.5* and ZsYellow may be due to the higher stability of reporter proteins from the transgene. Furthermore, the *etv2^+^* and *scl^+^* PAA angioblasts located in the ventral root of each sprout, and *nkx2.5* transcripts were transiently enriched in the clusters undergoing progenitor-to-angioblast transition (Fig. 1C,D). Taken together, these results show that the pharyngeal mesoderm is composed of distinct subpopulations with or without *nkx2.5* expression.

The above findings raised an interesting question about whether these subpopulations in the pharyngeal mesoderm undergo distinct cell fates. To answer this question, we performed lineage-tracing analysis in *Tg(nkx2.5:kaede)* embryos, where the pharyngeal mesodermal cells expressing photo-convertible Kaede proteins that could instantly switch from green to red fluorescence following ultraviolet light exposure (Guner-Ataman et al., 2013). In the first set of experiments, the Kaede proteins in the PAA progenitor cluster 3 and the subsequent posterior pharyngeal mesoderm located on the right-side of the embryo were photoconverted at 22 hpf, whereas the pharyngeal mesoderm on the left side remained unconverted as an internal control (Fig. 1E). As expected, the derivatives of the photoconverted cells were found in the sprouts of PAAs 3-5 at 36 hpf and in the endothelium of the aortic arches 3-6, as well as the ventral aorta at 60 hpf (Fig. 1F,G). Interestingly, less red fluorescence and more green fluorescence were observed in the cells of caudal PAAs 5 and 6, and the posterior region of ventral aorta (Fig. 1G). Nevertheless, these results indicate that the endothelial cells of PAAs and ventral aorta originate from the Kaede^+^ pharyngeal mesoderm.

Next, we specifically photoconverted the Kaede proteins in PAA cluster 5 at 36 hpf and found their red derivatives in PAA 5 at 54 hpf, but not in other PAAs (Fig. 1H,I). A few cells with red fluorescence were observed in the junction of PAA 5 and ventral aorta (Fig. 1I), suggesting the occurrence of endothelial cell rearrangements during blood vessel fusion (Herwig et al., 2011). In contrast, the photoconversion of Kaede^+^ cells located between PAA cluster 4 and 5 led to red derivatives housed specifically in ventral aorta (Fig. 1J,K). Based on these observations, we concluded that the pharyngeal mesoderm cells within PAAs 3-6 are specified into two vascular progenitor subpopulations: *nkx2.5^+^* cells that give rise to PAAs and *nkx2.5^−^* cells that generate the connective ventral aorta.

### Pharyngeal pouches have an essential role in PAA progenitor specification

**Fig. 2.**
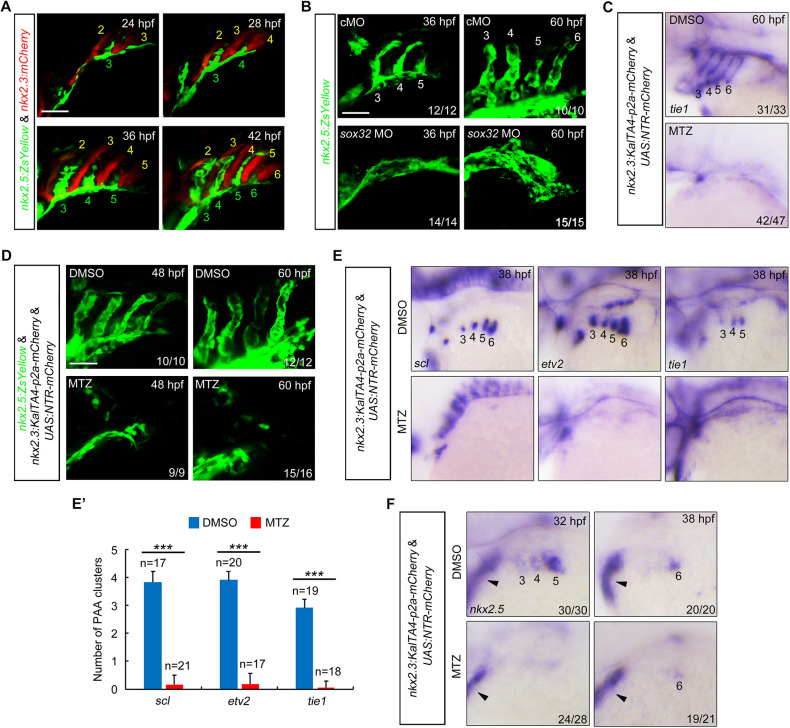
**Ablation of pouch endoderm impairs PAA progenitor specification.** (A) Confocal images of live *Tg(nkx2.5:ZsYellow;nkx2.3:mCherry)* embryos. The formation of mCherry^+^ pharyngeal pouches (red) coincides with the emergence of ZsYellow^+^ PAA clusters (green). Pharyngeal pouches and PAA clusters or sprouts are numbered with yellow or green numbers, respectively. Scale bar: 50 μm. (B) Confocal images of live *Tg(nkx2.5:ZsYellow)* embryos showed that injection of 8 ng *sox32* MO resulted in complete absence of PAA sprouts and PAA tubular structures. The ratios of affected embryos are indicated. Scale bar: 50 μm. (C,D) Embryos were exposed to 10 mM MTZ from bud stage to 38 hpf, and then harvested at the indicated developmental stages for *in situ* hybridization (C) or *in vivo* confocal imaging (D). Numbers in C indicate PAA3-PAA6. Scale bar: 50 μm. (E,E′) The *scl*, *etv2* and *tie1* transcripts were evaluated by *in situ* hybridization in *Tg(nkx2.3:KalTA4-p2a-mCherry;UAS:NTR-mCherry)* embryos treated with DMSO or 10 mM MTZ (E). The average numbers of *scl^+^*, *etv2^+^* and *tie1^+^* PAA angioblast clusters were quantified from three independent experiments and the group values are expressed as mean±s.d. (E′). Student's *t*-test, ***P<0.001. (F) Expression analysis of *nkx2.5* in pouch endoderm-depleted embryos. Black arrowheads represent the expression of *nkx2.5* in the developing heart.

We next aimed to determine the requirement of pouch endoderm during PAA morphogenesis. First, time-lapse recordings of pouch development and PAA formation were performed in *Tg(nkx2.3:mCherry;nkx2.5:ZsYellow)* embryos, where pouches were labeled with red fluorescent protein mCherry (Choe et al., 2013). At around 24 hpf, the third pharyngeal pouch appeared to have fully formed and reached the sprouting ZsYellow^+^ cluster 3 (Fig. 2A). At later stages, the fourth, fifth and sixth pouches successively made contact with the developing ZsYellow^+^ clusters for PAAs 4-6 (Fig. 2A), indicating a close interaction between the endodermal pouches and the progenitor clusters. Depletion of *sox32* in zebrafish embryos by injection antisense morpholinos (MOs) resulted in a lack of early endoderm and endoderm pouches (Fig. S2A,B) (Alexander et al., 1999). As previously described in endoderm-less *bon* mutants (Paffett-Lugassy et al., 2013), the ZsYellow^+^ cells remained in the pharyngeal region in *Tg(nkx2.5:ZsYellow)* embryos injected with *sox32* MO, but the PAAs were completely absent (Fig. 2B).

In order to examine the specific function of pouch endoderm in the establishment of PAAs, we generated a NTR-mediated tissue-ablation system, *Tg(nkx2.3:KalTA4-p2a-mCherry;UAS:NTR-mCherry)*, using an optimized Gal4-UAS system to drive NTR protein expression in *nkx2.3*^+^ cells (Curado et al., 2007, 2008; Distel et al., 2009). The *Tg(nkx2.3:KalTA4-p2a-mCherry;UAS:NTR-mCherry)* embryos were exposed to MTZ from the bud stage to 36 hpf. Live embryo imaging revealed that mCherry-expressing pouch endoderm was markedly reduced at 24 hpf and all the pouch structures were successfully ablated at 36 hpf (Fig. S3A). Intriguingly, in the pouch endoderm-ablated embryos, the expression of PAA endothelial cell marker *tie1* was absent and the ZsYellow*^+^* cells did not undergo sprouting nor give rise to PAAs (Fig. 2C,D). These results provide strong evidence that pharyngeal pouches are essential for PAA morphogenesis. In addition, pouch-depleted *Tg(flk:EGFP)* embryos also showed severe defects in these vessels (Fig. S3B), indicating the lack of plasticity in the formation of the PAAs (Nagelberg et al., 2015). Cxcl12b, a Cxcr4a ligand derived from the endoderm underlying the lateral dorsal aortae (LDA), has been reported to be required for the formation of LDA (Siekmann et al., 2009). Interestingly, the LDA in pouch endoderm-less embryos displayed no obvious malformations (Fig. S3B), suggesting the specificity of NTR-mediated pouch endoderm ablation in our related experiments.

To determine whether pharyngeal pouches function in PAA progenitor specification, the expression of putative earlier angioblast lineage markers *scl* and *etv2* and more mature angioblast marker *tie1* was first examined in MTZ-treated *Tg(nkx2.3:KaTAa4-p2a-mCherry;UAS:NTR-mCherry)* embryos at 38 hpf. We found that the formation of these angioblast clusters was abolished in embryos treated with MTZ (Fig. 2E,E′). We further generated a transgenic line, *Tg(sox10:KalTA4-p2a-mCherry)*, in which the red fluorescence of mCherry proteins was expressed in the neural crest cells. We then crossed fishes to generate *Tg(sox10:KalTA4-p2a-mCherry;UAS:NTR-mCherry)* embryos. MTZ treatment from the bud stage induced obvious cell death in the pharyngeal neural crest cells at 36 hpf (Fig. S3C). However, the PAA angioblasts developed normally (Fig. S3D), implying that pharyngeal neural crest cells are not necessary for the specification and angioblast differentiation of PAA progenitors. We then analyzed the expression of the PAA progenitor marker *nkx2.5* in pouch-depleted embryos and found a significant reduction of *nkx2.5*-expressing progenitors of PAAs 3-6 (Fig. 2F). Moreover, at 18 hpf, pouch depletion did not disrupt the segregation of the *nkx2.5^+^* common progenitors (Fig. S4A,B) (Guner-Ataman et al., 2018). Taken together, these results suggest that pouches are essential for PAA progenitor specification in the pharyngeal region.

### Pharyngeal pouches provide a niche for BMP signal activation in presumptive PAA progenitors

**Fig. 3.**
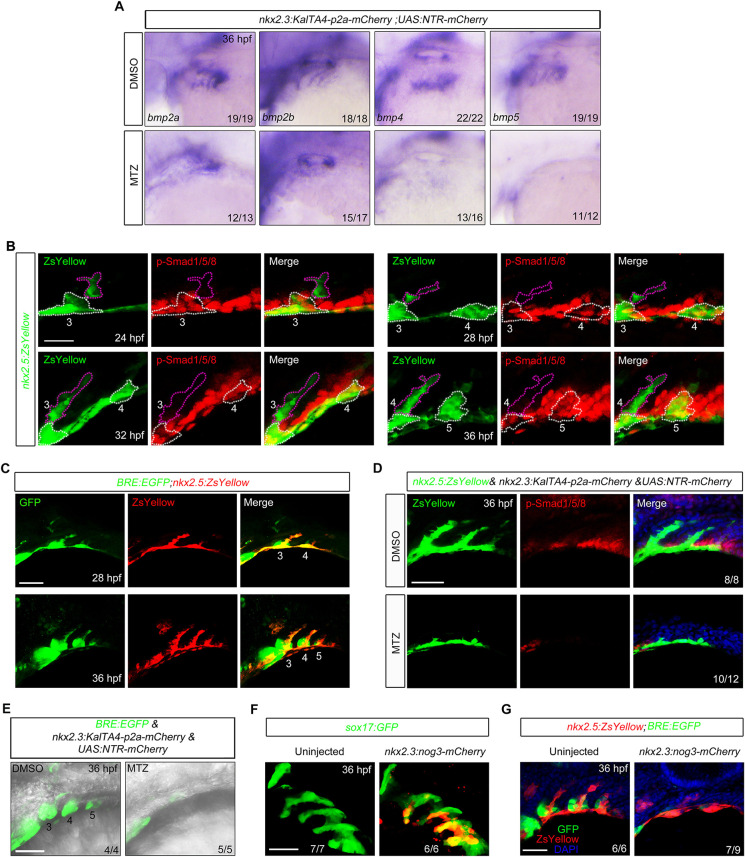
**Pouch endoderm is necessary for BMP signal activation in the presumptive PAA progenitors.** (A) The *bmp2a*, *bmp2b*, *bmp4* and *bmp5* transcripts were evaluated by *in situ* hybridization in *Tg(nkx2.3:KalTA4-p2a-mCherry*;*UAS:NTR-mCherry)* embryos treated with DMSO or 10 mM MTZ. (B) BMP signal was dynamically activated in the forming PAA clusters. *Tg(nkx2.5:ZsYellow)* embryos were harvested at indicated stages and subjected to immunostaining for p-Smad1/5/8 (red) and ZsYellow (green). The white dotted lines outline the PAA progenitor clusters and the purple dotted lines indicate the PAA sprouts composed of migrating angioblasts. Scale bar: 20 μm. (C) *Tg(BRE:EGFP;nkx2.5:ZsYellow)* embryos were immunostained for GFP (green) and ZsYellow (red) to visualize BMP-responsive cells and PAA clusters. Scale bar: 50 μm. (D) p-Smad1/5/8 levels were greatly decreased in pouch endoderm-depleted embryos. *Tg(nkx2.5:ZsYellow;nkx2.3:KalTA4-p2a-mCherry;UAS:NTR-mCherry)* embryos were treated with DMSO or 10 mM MTZ from bud stage to 36 hpf, and then stained for p-Smad1/5/8 (red) and ZsYellow (green). Scale bar: 50 μm. (E) Live confocal images of *Tg(BRE:EGFP;nkx2.3:KalTA4-p2a-mCherry;UAS:NTR-mCherry)* embryos treated with DMSO or 10 mM MTZ from bud stage to 36 hpf. Scale bar: 50 μm. (F) *Tg(sox17:GFP)* embryos were injected with 40 pg *nkx2.3:noggin3-mCherry* plasmids and 200 pg Tol2 transposase mRNA at the one-cell stage, and then harvested for *in vivo* confocal imaging to visualize pharyngeal pouches (green) and the expression of Noggin3-mCherry fusion proteins (red). Scale bar: 50 μm. (G) Pouch-derived Noggin3 significantly decreased the BMP signal activity in the pharyngeal region. *Tg(nkx2.5:ZsYellow;BRE:EGFP)* embryos were injected with 40 pg *nkx2.3:noggin3-mCherry* plasmids and 200 pg Tol2 transposase mRNA at the one-cell stage, and then embryos with abundant mCherry fluorescence in the pouches were selected at 36 hpf for immunostaining. Scale bar: 50 μm.

Several genes encoding BMP ligands, including *bmp2a*, *bmp2b*, *bmp4* and *bmp5*, are expressed in the pouch endoderm during pharyngeal segmentation (Holzschuh et al., 2005). Indeed, when pouch endoderm was ablated, expression of these BMP genes were eliminated in the pharyngeal region (Fig. 3A). Immunostaining experiments revealed robust signals of phosphorylated Smad1, Smad5 and Smad8 (Smad1/5/8), the intracellular effectors for BMP signaling, in the forming ZsYellow^+^ clusters and neighboring cranial neural crest cells of *Tg(nkx2.5:ZsYellow)* embryos (Fig. 3B). In contrast, no detectable phosphorylation of Smad1/5/8 could be seen in the ZsYellow^+^ sprouts composed of the migrating PAA angioblasts (Fig. 3B), suggesting the activation of canonical BMP signaling occurs primarily in the early stage of PAA morphogenesis. Additionally, we crossed *Tg(nkx2.5:ZsYellow)* with *Tg(BRE:EGFP)*, a BMP signal activity reporter line (Laux et al., 2011). We observed strong GFP protein expression in the ZsYellow^+^ clusters and the other nearby tissues (Fig. 3C). These results demonstrate that BMP/Smad signal is highly activated in the presumptive PAA progenitors.

We then ablated the pharyngeal pouches in *Tg(nkx2.5:ZsYellow)* and *Tg(BRE:EGFP)* embryos, respectively. Interestingly, the pouch depletion led to an evident decrease in BMP signal activity in both ZsYellow^+^ clusters and other pharyngeal tissues (Fig. 3D,E). These findings imply that pharyngeal pouches function as a niche for activating BMP signal in presumptive PAA progenitors. To further confirm this assumption, *nkx2.3:noggin3-mCherry* plasmids expressing a secreted BMP inhibitory protein Noggin3 (Ning et al., 2013), along with Tol2 transposase mRNA, were co-injected into *Tg(sox17:GFP)* embryos. Part of the injected embryos exhibited uneven, but abundant, mCherry fluorescence in the pouches (Fig. 3F). The same injections were then performed in *Tg(nkx2.5:ZsYellow;BRE:EGFP)* embryos. The embryos with strong mCherry fluorescence in the pharyngeal region were selected at 36 hpf for immunostaining analysis. The pouch-derived Noggin3 had no obvious effect on pouch endoderm development, as indicated by *nkx2.3* expression, but significantly inhibited the BMP signal activity in pharyngeal region (Fig. 3G, Fig. S5), indicating that the secreted BMP ligands from pouches are the biochemical niche cues that trigger signal activation in presumptive PAA progenitors.

### BMP signaling is required for PAA progenitor specification

**Fig. 4.**
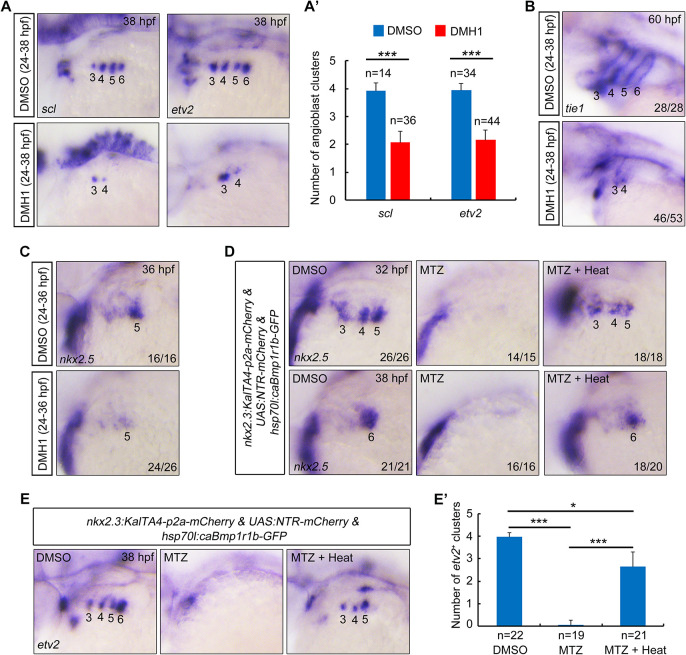
**Inhibition of BMP signaling impairs PAA progenitor specification.** (A,A′) Transcripts of *scl* and *etv2* were evaluated by *in situ* hybridization in DMSO- or DMH1-treated embryos. Embryos were treated with DMSO or 10 μM DMH1 from 24 to 38 hpf. Numbers indicate PAA angioblast clusters. The average numbers of *scl^+^* and *etv2^+^* PAA angioblast clusters are quantified in A′ based on three independent experiments and the group values are expressed as mean±s.d. Student's *t*-test, ***P<0.001. (B,C) Wild-type embryos were treated with DMSO or with 10 μM DMH1 within the indicated time windows. Subsequently, these embryos were harvested for *in situ* hybridization. The numbers indicate the PAAs (B) or PAA progenitor clusters (C). (D-E′) Ectopic induction of caBmpr1b rescues the defects of PAA progenitor specification and angioblast differentiation in the pouch-depleted embryos. *Tg(nkx2.3:KalTA4-p2a-mCherry;UAS:NTR-mCherry;hsp70l:caBmpr1b-GFP)* embryos were treated with 10 mM MTZ from bud stage, heat shocked at 24 hpf for 20 min, and then harvested at the indicated stages for *in situ* hybridizations with *nkx2.5* (D) and *etv2* (E) probes. The average numbers of *etv2^+^* PAA angioblast clusters were quantified from three independent experiments (E′). Significance of differences compared with MTZ treatment group were analyzed with Student's *t*-test (*P<0.05; ***P<0.001).

To determine whether BMP signaling is required for PAA progenitor specification, we first examined the expression of angioblast marker genes *scl* and *etv2* in embryos treated with DMH1, a selective chemical inhibitor of the BMP pathway (Hao et al., 2010, 2014). Interestingly, embryos treated with DMH1 from 20 hpf exhibited impaired angioblast formation in PAA clusters 4-6, whereas the angioblasts in cluster 3 developed normally (Fig. S6A,A′). To further elucidate the role of the BMP pathway in angioblast formation, we treated embryos with DMH1 from 16 hpf, when the common progenitors of PAAs, HM and cardiac OFT have been specified in the ALPM (Guner-Ataman et al., 2018). Noticeably disturbed generation of angioblast clusters 3 and 4-6 was observed (Fig. S6B). However, blocking BMP signaling from such early stages (16 and 20 hpf) led to different severities of pouch defects (Fig. S6C), which contributed to the difficulties in distinguishing a direct role of BMP pathway in PAA development. Fortunately, we found that embryos treated with DMH1 from 24 hpf, preceding the formation of PAA cluster 4, showed normal pouch structures (Fig. S6C). However, the expression of *scl* and *etv2* was decreased in cluster 4 and completely abolished in clusters 5 and 6 (Fig. 4A,A′). Hereafter, dorsomorphin, another small chemical inhibitor of BMP signaling (Yui et al., 2008), was applied to wild-type embryos from 24 hpf and resulted in similar angioblast phenotypes (Fig. S7A,A′). Consistent with these findings, blocking BMP signaling from 24 hpf greatly reduced the expression of endothelial cell marker *tie1* in the caudal PAAs at 60 hpf (Fig. 4B). Similar angioblast and PAA defects were observed in *Tg(hsp70l:dnBmpr1a-GFP)* embryos that were heat-shocked at 24 hpf (Fig. S7B,C), excluding the potential off-target effects of the pharmacological treatments.

Next, we analyzed the expression of PAA progenitor marker *nkx2.5* in BMP signal-suppressed embryos at 36 hpf. BMP signal inhibition from 24 hpf significantly repressed the *nkx2.5* expression (Fig. 4C), indicating a serious imperfection of PAA progenitor specification. Unexpectedly, when *Tg(hsp70l:caBmpr1b-GFP)* embryos were heat shocked at 24 hpf to induce the expression of constitutively active BMP receptor 1b (caBmpr1b), the phosphorylation of Smad1/5/8 was evidently elevated, while PAA progenitor specification and angioblast differentiation remained unchanged (Fig. S7D-G). These results suggest that BMP signal activation is necessary, but not sufficient, for PAA progenitor specification. Furthermore, the pouch depletion-induced deficiencies in PAA progenitor specification and subsequent angioblast differentiation were recovered in heat-shocked *Tg(hsp70l:caBmpr1b-GFP)* embryos (Fig. 4D-E′). However, these defects in MTZ-treated embryos without the *hsp70l:caBmpr1b-GFP* transgene were not alleviated by heat-shock treatment (Fig. S8A,B), ruling out the inactivating effects of heat-shock on MTZ in the pouch-depletion experiments. These results imply that pharyngeal pouches induce PAA progenitor specification via activation of BMP signaling in the pharyngeal mesoderm.

### BMP signaling is dispensable for angioblast differentiation, dorsal migration, endothelial maturation and lumen formation during PAA morphogenesis

**Fig. 5.**
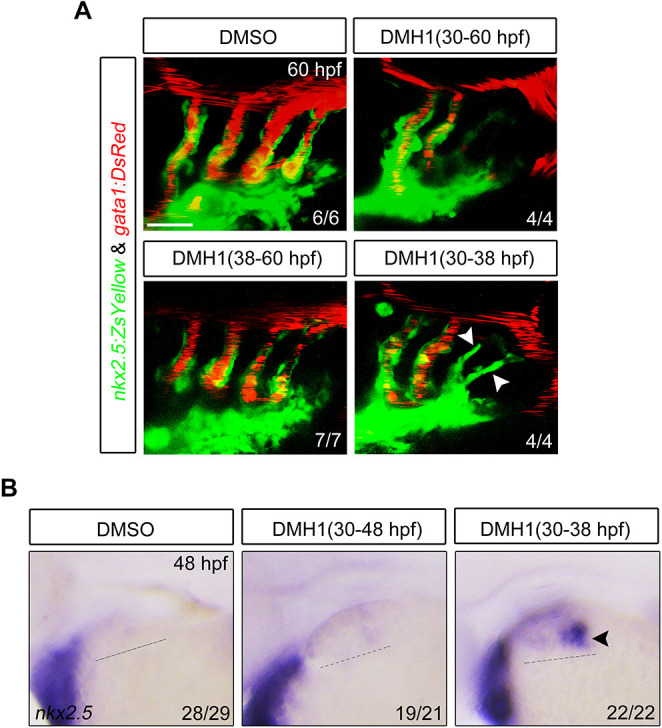
**Inhibition of BMP signaling after PAA progenitor specification induces no detectable defects of PAA morphogenesis.** (A) Live confocal images of *Tg(nkx2.5:ZsYellow;gata1:DsRed)* embryos with DMSO or 10 μM DMH1 treatment for different durations. White arrowheads indicate the new sprouting clusters after removal of DMH1 at 38 hpf. Scale bar: 50 μm. (B) Expression analysis of *nkx2.5* by *in situ* hybridization in embryos subjected to different treatments. The dotted lines represent the area of expression of *nkx2.5*. Black arrowhead indicates the newly emerged expression of *nkx2.5* after removal of DMH1.

To explore whether BMP signaling has a role in angioblast differentiation, DMH1 treatment was performed in *Tg(nkx2.5:ZsYellow;gata1:DsRed)* embryos between 30 and 60 hpf, a time window after the specification of progenitors for PAAs 3 and 4. Such DMH1 treatment abolished the formation of PAAs 5 and 6, and led to a lack of blood flow in these caudal PAAs, but had no obvious impact on PAAs 3 and 4 (Fig. 5A). When the DMH1 treatment was carried out from 38 hpf, a time point when most of the *nkx2.5*^+^ progenitors had accomplished angioblast transition, no obvious defects in PAA development were observed in the resulting embryos (Fig. 5A). These results indicate that BMP signaling is crucial for progenitor specification, while dispensable for angioblast differentiation, dorsal migration, endothelial maturation and lumen formation during PAA morphogenesis. It is interesting that if the DMH1 treatment was performed from 30 hpf and then terminated 8 h later, *nkx2.5*^+^ progenitors for the caudal PAAs reappeared at 48 hpf and went on to develop into growing sprouts at 60 hpf (Fig. 5A,B). These observations imply that, when the BMP inhibition is removed, BMP signal might be reactivated in the pharyngeal mesoderm cells and restore the formation of PAA progenitors.

### BMP2a and BMP5 function together in PAA progenitor specification

**Fig. 6.**
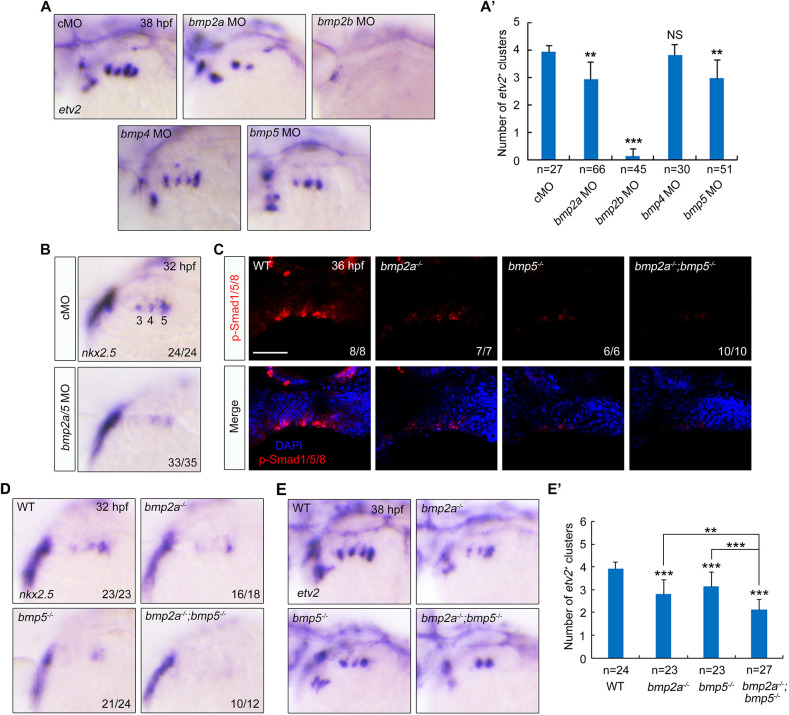
**BMP2a together with BMP5 functions in PAA progenitor specification.** (A,A′) The expression of *etv2* in embryos injected with indicated MO was analyzed by *in situ* hybridization (A). Injection doses: *bmp2a* MO, 2 ng; *bmp2b* MO, 0.3 ng; *bmp4* MO, 2 ng; *bmp5* MO, 4 ng. The average numbers of *etv2^+^* PAA angioblast clusters were quantified from three independent experiments, and the group values are expressed as mean±s.d. (A′). Student's *t*-test (**P<0.01, ***P<0.001; NS, no significant difference). (B) Knockdown of *bmp2a* and *bmp5* disrupted the specification of PAA progenitors. Wild-type embryos were injected with *bmp2a* and *bmp5* MOs at the one-cell stage. The resulting embryos were harvested for *in situ* hybridization. (C) Wild-type and indicated mutant embryos were harvested at 36 hpf for immunofluorescence assay with anti-p-Smas1/5/8 antibody. Nuclei were counterstained with DAPI. There is a distinct decrease of p-Smad1/5/8 in the *bmp2a^−/−^;bmp5^−/−^* double mutants. Scale bar: 50 μm. (D-E′) Expression analysis of *nkx2.5* (D) and *etv2* (E) in *bmp2a^−/−^* or *bmp5^−/−^* embryos and *bmp2a^−/−^;bmp5^−/−^* double mutants by *in situ* hybridization. The average numbers of *etv2^+^* angioblast clusters were quantified from three independent experiments and the group values were expressed as mean±s.d. (E′). Student's *t*-test was used to determine the significance of differences between wild-type animals and each mutant, and one-way ANOVA test was performed to analyze the statistical differences between *bmp2a^−/−^;bmp5^−/−^* double mutants and *bmp2a^−/−^* or *bmp5^−/−^* embryos. **P<0.01, ***P<0.001.

To explore which BMP ligands are specifically required for PAA progenitor specification, knockdown experiments were performed using previously validated antisense MOs targeting *bmp2a*, *bmp2b*, *bmp4* and *bmp5* (Chocron et al., 2007; Li et al., 2019; Naye et al., 2012; Shih et al., 2017). As expected, injection of these MOs into wild-type embryos caused clear defects in the development of the hepatic bud, pharyngeal pouches, presumptive cloaca and neural crest cells, respectively (Fig. S9A-D) (Li et al., 2019; Naye et al., 2012; Shih et al., 2017; Stickney et al., 2007), indicating a satisfactory level of efficiency and specificity of these MOs. We observed that the expression of *etv2* was not obviously changed in embryos injected with *bmp4* MO (Fig. 6A,A′). However, *etv2* expression was almost abolished in *bmp2b* morphants (Fig. 6A,A′). We have previously reported that *bmp2b* is essential for pharyngeal pouch progenitor specification (Li et al., 2019). In fact, *bmp2b* morphants showed no pharyngeal pouches at 36 hpf, as indicated by the expression of the pouch epithelium marker *nkx2.3* (Fig. S9B). Therefore, although we cannot rule out the possibility that *bmp2b* plays a direct role in PAA development, the loss of PAA angioblast in *bmp2b* morphants is due mainly to the deficiency of pharyngeal pouches. Importantly, knockdown of *bmp2a* or *bmp5* resulted in a steady reduction in the number of *etv2^+^* clusters (Fig. 6A,A′). Furthermore, the expression of *nkx2.5* was evidently decreased in the pharyngeal region of embryos injected with *bmp2a* and *bmp5* MOs (Fig. 6B). Together, these data suggest that *bmp2a* and *bmp5* may play an important role in PAA progenitor specification.

To examine the direct function of pouch-expressed BMP ligands, we performed tissue-specific knockdown experiments using a KalTA4-UAS system to drive the expression of miR30-based short hairpin RNAs (shRNAs), which is widely used for gene silencing in eukaryotic organisms (Fig. S10A) (Li et al., 2018; Stegmeier et al., 2005; Zeng et al., 2005). We first generated an *UAS:EGFP-shRNA* plasmid expressing a shRNA targeting *bmp2a* (named *shRNA-bmp2a*). *KalTA4-p2a-mCherry* mRNA and *shRNA-bmp2a* were co-injected into one-cell stage embryos, and the expression of *bmp2a* was examined by whole-mount *in situ* hybridization and quantitative real-time PCR. KalTA4-mediated expression of *shRNA-bmp2a* clearly knocked down endogenous *bmp2a* expression (Fig. S10B,B′). Similarly, we found that *shRNA-bmp2b*, *shRNA-bmp4* and *shRNA-bmp5* could evidently silence genes when employed independently (Fig. S10C-E′). Furthermore, these *shRNA-*mediated gene knockdowns led to similar defects to those found in the related mutants or morphants (Fig. S10F-I) (Li et al., 2019; Naye et al., 2012; Shih et al., 2017; Stickney et al., 2007). These analyses provide further evidence for the efficiency of these shRNAs.

Next, these *UAS:EGFP-shRNA* plasmids and Tol2 transposase mRNA were injected into *Tg(nkx2.3:KalTA4-p2a-mCherry)* embryos. A subset of the resulting embryos showed bright-green fluorescence in the pharyngeal pouches at 36 hpf as previously reported (Fig. S11A) (Li et al., 2018). Such embryos were collected to examine the developmental consequences of BMP gene deficiency. We found that these shRNAs selectively disturbed the expression of their target genes in the pouches (Fig. S11B-E). Interestingly, although the depletion of *bmp2b* or *bmp4* expression in pouches had no effect on angioblast formation, silencing of *bmp2a* or *bmp5* eliminated the expression of *etv2* in the pharyngeal region (Fig. S11F,F′). Moreover, knockdown of both *bmp2a* and *bmp5* reduced the expression of *nkx2.5* in the PAA clusters, but did not affect the formation of pharyngeal pouches (Fig. S11G,H). These findings support the idea that pouch-derived BMP2a and BMP5 are responsible for PAA progenitor specification.

To further substantiate the function of *bmp2a* and *bmp5* in PAA development, we generated one genetic mutant line for each gene using CRISPR/CAS9 technology. The mutant allele of *bmp2a* or *bmp5* carries a DNA deletion near the gRNA targeting sequence in the first exon, resulting in a premature stop codon and presumably a truncated protein lacking the prodomain and C-terminal mature peptide (Fig. S12A,B). *In situ* hybridization results revealed that about 50% of *bmp2a^−/−^* and *bmp5^−/−^* mutants, which were confirmed by genotyping, showed defective development of the hepatic bud or neural crest cells (Fig. S12C,D). However, there was no compensational increase in the expression of *bmp2a* or *bmp5* in relevant mutants (Fig. S12E,F), suggesting that the incomplete penetrance is not due to compensatory functions between these two genes.

The *bmp2a^−/−^* and *bmp5^−/−^* embryos exhibited no apparent morphological defects and could live to adulthood. We then generated the *bmp2a^−/−^;bmp5^−/−^* double mutant by incrossing *bmp2a^−/−^* and *bmp5^−/−^* fishes. A small proportion (about 20%) of *bmp2a^−/−^;bmp5^−/−^* embryos displayed evident pericardial edema and died before 7 days post-fertilization, but the rest showed no gross morphological and survival differences compared with wild-type embryos. To confirm the role of *bmp2a* and *bmp5* in PAA progenitor specification, immunostaining analysis was first performed in these mutants at 36 hpf. As shown in Fig. 6C, compared with wild-type embryos, the phosphorylation level of Smad1/5/8 in the pharyngeal region was decreased in *bmp2a^−/−^* and *bmp5^−/−^* mutants, and almost abolished in *bmp2a^−/−^*;*bmp5^−/−^* embryos. Besides, these mutants exhibited obviously impaired formation of PAA progenitors (Fig. 6D). Finally, when compared with control animals and *bmp2a^−/−^* or *bmp5^−/−^* embryos, a significant reduction in *etv2*^+^ angioblast clusters was observed in *bmp2a^−/−^;bmp5^−/−^* double mutants, while the pharyngeal pouches were normally developed (Fig. 6E,E′, Fig. S12G).

Collectively, these data suggest that, among the BMPs expressed in the pouch endoderm, BMP2a and BMP5 are crucial for BMP pathway activation in the pharyngeal mesoderm, thereby promoting PAA progenitor specification.

### *npas4l* is expressed in PAA progenitors in the pharyngeal region

**Fig. 7.**
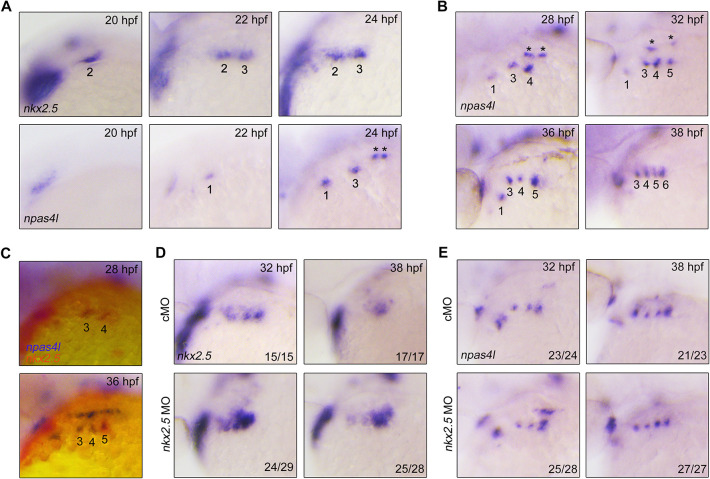
***npas4l* is expressed in both PAA progenitors and angioblasts.** (A,B) Analysis of the expression patterns of *nkx2.5* and *npas4l* in the pharynx at the indicated stages by *in situ* hybridization. The asterisks indicate the expression of *npas4l* in the lateral dorsal aortae. (C) Double *in situ* hybridization of *npas4l* (red) and *nkx2.5* (blue) expression at 28 and 36 hpf. (D,E) Expression analysis of *nkx2.5* (D) and *npas4l* (E) in embryos injected with 3 ng *nkx2.5* MO. The expression of *nkx2.5* but not *npas4l* was clearly increased in *nkx2.5* morphants.

The progenitors for PAA, HM and cardiac OFT are all marked by *nkx2.5* expression (Guner-Ataman et al., 2018; Paffett-Lugassy et al., 2013). Future identification of specific biomolecular markers for PAA progenitors can provide new avenues to investigate the cellular and molecular events in PAA progenitor specification. A recent study identified *npas4l*, which encodes a PAS-domain-containing bHLH transcription factor, as the gene defective in the *cloche* mutant that lacks most endothelial as well as hematopoietic cells (Reischauer et al., 2016). Henceforth, *npas4l* is also called *cloche*. Therefore, we speculate that *npas4l* may be expressed in PAA progenitors and be crucial for PAA development. To verify this hypothesis, the expression of *npas4l* in the pharyngeal region was analyzed by *in situ* hybridization. We found that *npas4l* was not expressed in the pharynx at 20 hpf (Fig. 7A). However, *npas4l* transcripts were then detected in the presumptive PAA progenitor cluster 3 at 24 hpf, ∼2 h later than the initial expression of *nkx2.5* in the same PAA cluster (Fig. 7A). Over the next 14 h, *npas4l* transcripts gradually appeared in a craniocaudal sequence in the PAA clusters (Fig. 7B). Moreover, the expression of *npas4l* in the PAA clusters was further confirmed by the colocation of *npas4l* and *nkx2.5* transcripts (Fig. 7C).

A previous study has shown that the expression of *nkx2.5* is reduced following the differentiation of PAA progenitors into angioblasts (Paffett-Lugassy et al., 2013). Intriguingly, *npas4l* transcripts persisted during PAA progenitor differentiation (Fig. 7B). These observations raised the possibility that *npas4l* is not only expressed in the progenitors but also in the angioblasts of PAAs. It has been shown that injection of *nkx2.5* MO into zebrafish embryos can disrupt the angioblast differentiation and result in an accumulation of PAA progenitors (Paffett-Lugassy et al., 2013). We then examined the expression of *npas4l* in *nkx2.5* morphants. If *npas4l* is specifically expressed in PAA progenitors, we would expect a clear increase of *npas4l* expression in *nkx2.5* morphants. Indeed, compared with control animals, embryos injected with *nkx2.5* MO showed much higher levels of *nkx2.5* expression (Fig. 7D). By contrast, the expression levels of *npas4l* were not obviously changed in the pharynx upon *nkx2.5* MO injection (Fig. 7E). These results may imply that, although the inhibition of *nkx2.5* function led to excess PAA progenitors at the expense of angioblasts, the total number of cells with endothelial potential was unchanged. Thus, *npas4l* is expressed in both PAA progenitors and angioblasts.

### *npas4l* is essential for endothelial lineage progression from the pharyngeal mesoderm to PAA progenitors

**Fig. 8.**
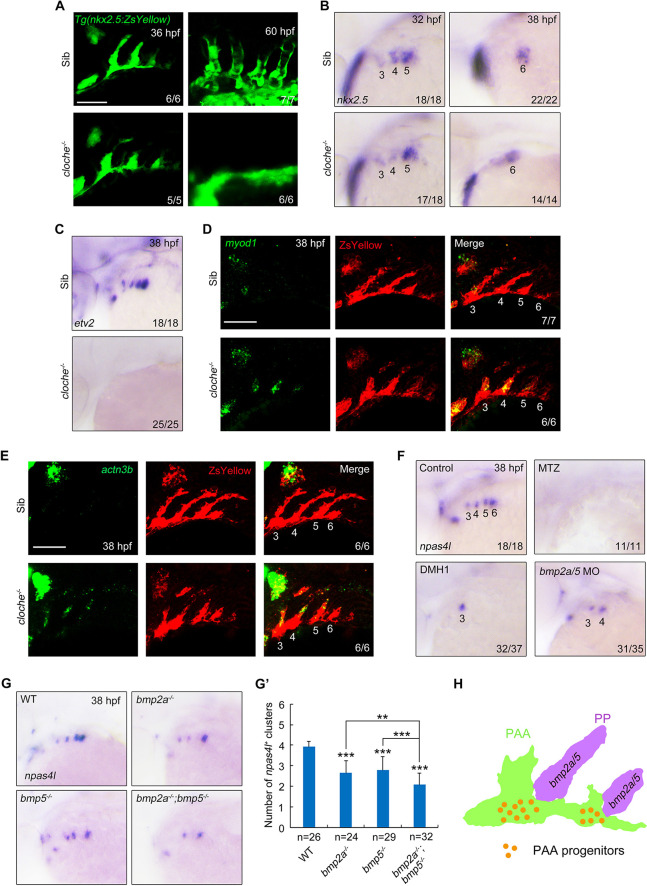
***npas4l* plays a pivotal role in the specification of PAA progenitors.** (A) Live confocal images of *npas4l^−^*^/−^ mutants and their wild-type and heterozygous siblings in *Tg(nkx2.5:ZsYellow)* background*.* Scale bar: 50 μm. The *npas4l* gene is also called *cloche*. (B,C) The expression patterns of *nkx2.5* (B) and *etv2* (C) were analyzed in *npas4l^−/−^* mutants and their siblings by *in situ* hybridization. (D,E) The expression of *myod1* (D) and *actn3b* (E) in the pharynx were analyzed in *npas4l^−/−^* mutants and their siblings in *Tg(nkx2.5:ZsYellow)* background. The embryos were first subjected to fluorescent *in situ* hybridization with *myod1* or *actn3b* probe, and then stained with anti-ZsYellow antibody. (F) *Tg(nkx2.3:KalTA4-p2a-mCherry;UAS:NTR-mCherry)* embryos were treated with 10 mM MTZ from bud stage and wild-type embryos were treated with 10 μM DMH1 from 24 hpf or injected with 2 ng *bmp2a* MO together with 4 ng *bmp5* MO at the one-cell stage. The resulting embryos were harvested for *in situ* hybridization with *npas4l* probe at 38 hpf. (G,G′) Expression analysis of *npas4l* in *bmp2a^−/−^* or *bmp5^−/−^* single mutants and *bmp2a^−/−^;bmp5^−/−^* double mutant embryos (G). The average numbers of *npas4l^+^* clusters were quantified from three independent experiments and the group values are expressed as mean±s.d. (G′). Student's *t*-test was used to determine the significance of differences between wild-type animals and indicated mutants, and one-way ANOVA test was performed to analyze the statistical differences between *bmp2a^−/−^;bmp5^−/−^* embryos and *bmp2a^−/−^* or *bmp5^−/−^* single mutants. **P<0.01, ***P<0.001. (H) Working model depicting that the specification of PAA progenitors from pharyngeal mesoderm is dependent of the activation of BMP signaling by *bmp2a* and *bmp5* expressed in pouch endoderm.

We next examined whether *npas4l* plays a role in PAA development and observed that, in comparison with wild-type and heterozygous siblings, *cloche* homozygous (*cloche^−/−^*) mutants in *Tg(nkx2.5:ZsYellow)* background showed almost normal formation of ZsYellow^+^ clusters at 36 hpf (Fig. 8A). However, the PAA vascular channels were absent in *cloche^−/−^* embryos at 60 hpf (Fig. 8A). Interestingly, slightly different from our results, some residual PAA vasculatures were found in *cloche^−/−^* mutants expressing the *Tg(flk:EGFP)* transgene (Reischauer et al., 2016). As endothelial cells from the LDA could compensate for the loss of PAA vessels under certain conditions (Nagelberg et al., 2015), the *flk^+^* PAA endothelial cells in *cloche^−/−^* mutants might be due to the plasticity during PAA development.

Our *in situ* hybridization analyses further revealed that, in comparison with wild-type and heterozygous siblings, *cloche* homozygous (*cloche^−/−^*) mutants exhibited normal *nkx2.5* expression in pharyngeal clusters 3-5 at 32 hpf (Fig. 8B). To our surprise, the expression of *etv2*, the PAA angioblast marker, was completely missing in the *cloche^−/−^* mutants at 38 hpf (Fig. 8C), suggesting an unsuccessful differentiation of *nkx2.5^+^* progenitors. This phenomenon thus raised an interesting question about the cell fate of the *nkx2.5^+^* progenitors in *cloche^−/−^* mutants. It has been suggested that the *nkx2.5^+^* progenitors in the lateral plate mesoderm can differentiate into various pharyngeal tissues, including PAA, HM and cardiac OFT (Guner-Ataman et al., 2018; Paffett-Lugassy et al., 2013). Therefore, we investigated whether the *nkx2.5^+^* progenitors within presumptive PAA clusters in *cloche^−/−^* mutants altered their cell fate to give rise to cardiac OFT and/or to become muscle cells. We found no distinct difference in the expression of *mef2cb* and *ltbp3*, both of which label the outflow pole of the heart tube (Zeng and Yelon, 2014; Zhou et al., 2011), between *cloche^−/−^* mutants and their siblings (Fig. S13A,B). On the contrary, the transcripts of the head muscle precursor marker *myod1* and the pharyngeal musculature marker *actn3b* were unexpectedly expressed in the presumptive PAA structures of *cloche^−/−^* mutants (Fig. 8D,E) (Holterhoff et al., 2009; Lin et al., 2006), suggesting a muscle cell fate transformation of the *nkx2.5^+^* progenitors. Therefore, before *npas4l* expression, the pharyngeal mesoderm seems to have multilineage differentiation potential. Together, these data suggest that *npas4l* plays a pivotal role in the specification of PAA progenitors from pharyngeal mesoderm.

To determine whether pharyngeal pouches are required for *npas4l* expression, *Tg(nkx2.3:KalTA4-p2a-mCherry;UAS:NTR-mCherry)* embryos were exposed to MTZ from the bud stage. Ablation of pouch endoderm completely abolished *npas4l* expression in the pharynx at 38 hpf (Fig. 8F). Moreover, both DMH1 treatment and injection with MOs targeting *bmp2a* and *bmp5* induced a dramatic reduction in *npas4l* transcripts (Fig. 8F). We also found a steady decrease in the number of *npas4l^+^* PAA clusters in *bmp2a^−/−^* or *bmp5^−/−^* single mutants and *bmp2a^−/−^;bmp5^−/−^* embryos (Fig. 8G,G′). Taken together, these findings support the idea that the pharyngeal pouches provide a niche microenvironment for the commitment of multipotent pharyngeal mesoderm toward PAA progenitors through expressing BMP2a and BMP5 (Fig. 8H).

## DISCUSSION

Improper embryonic development of the PAAs may cause life-threating congenital cardiovascular defects (Abrial et al., 2017; Srivastava, 2001). Malformations of the aortic arch system are often accompanied by anomalies of endodermal pouches, which lead to compromised pharyngeal segmentation (Jerome and Papaioannou, 2001; Lindsay et al., 2001; Piotrowski et al., 2003). The effects of pouches on aortic arch development have traditionally been considered secondary to pharyngeal patterning defects (Kopinke et al., 2006; Matt et al., 2003; Wendling et al., 2000). In this study, our results support a model in which the pharyngeal pouches provide a niche microenvironment for PAA progenitor specification via the expression of BMP proteins. Our findings suggest that the segmentation of pharyngeal pouches coincides spatiotemporally with the emergence of PAA progenitor clusters. Furthermore, depletion of pouch endoderm in zebrafish embryos by an MTZ-NTR system resulted in a remarkable reduction of BMP signal activity in the pharyngeal mesoderm and the complete absence of PAA structures attributed to impaired progenitor specification. Most importantly, the PAA progenitor specification is directly regulated by pouches and their derived signal molecules, as the ablation of pharyngeal neural crest cells shows no effect on the emergence of PAA angioblasts, which are differentiated from vascular progenitors. These data, combined with our recent findings that pharyngeal pouches regulate PAA angioblast proliferation by expressing PDGF ligands (Mao et al., 2019), suggest multiple distinct roles of pouch endoderm during PAA development.

It has been reported that a common progenitor population for PAAs, HM and cardiac OFT is specified in zebrafish ALPM during mid-somitogenesis (Guner-Ataman et al., 2018). At later stages, these progenitors, which are located within pharyngeal arches 3-6, contribute to the endothelium of their respective PAAs (Paffett-Lugassy et al., 2013). Interestingly, our data indicate that, at later stages, this pharyngeal mesoderm lineage within PAs 3-6 comprises two subpopulations: one of which is *nkx2.5^+^*, in which cells are restricted to different domains; the other is *nkx2.5^−^* , in which cells are located between the *nkx2.5^+^* clusters. Consistent with previous findings, our cell-lineage tracing analysis reveals that the *nkx2.5^+^* clusters sprout out and contribute to corresponding PAAs (Paffett-Lugassy et al., 2013). Previous reports have also suggested that, in zebrafish, the ventral parts of PAAs merge to form the bilateral ventral aortae (Anderson et al., 2008; Paffett-Lugassy et al., 2013). Unexpectedly, we found that the *nkx2.5^−^* subpopulation does not migrate dorsally and ultimately gives rise to ventral aortae. Therefore, PAAs are sequentially generated from *nkx2.5^+^* progenitors within the developing ventral aortae (the pharyngeal mesoderm). Interestingly, resin filling of mouse embryonic vasculature has shown that the PAA endothelium arises by branching off the aortic sac – the mammalian homolog of the ventral aorta of gill-bearing vertebrates (Berta, 2006; Hiruma et al., 2002). Based on these results, it is likely that the process of PAA morphogenesis is evolutionarily conserved across vertebrate classes.

Several lines of evidence support the idea that the pharyngeal mesoderm lineage is further specified into PAA progenitors in a niche microenvironment provided by pouches. First, the pharyngeal mesoderm within PAs 3-6 contains *nkx2.5^+^* progenitors that give rise to PAAs and *nkx2.5^−^* progenitors that generate ventral aorta. Second, the PAA progenitor clusters emerge in a craniocaudal sequence following pharyngeal pouch segmentation. Third, depletion of pouch endoderm eliminates the PAA progenitors without disrupting the segregation of pharyngeal mesoderm lineage from cardiac precursors. Fourth, BMP signal inhibition produced by pharmacological treatments and tissue-specific knockdown or genetic depletion of *bmp2a*/5 results in remarkably reduced PAA progenitors. Finally, yet most importantly, head muscle markers are ectopically expressed in the presumptive PAA structures of *cloche*^−/−^ mutants, implying that the pharyngeal mesoderm has multilineage differentiation potential. This idea is supported by a previous observation that, in *cloche^−/−^* mutants, the rostral mesoderm undergoes a fate transformation and generates ectopic cardiomyocytes (Schoenebeck et al., 2007).

Our study demonstrates that *npas4l* is essential for PAA progenitor specification and its expression is tightly controlled by BMP signaling. Moreover, mutation of the *cloche* locus results in a cell fate transformation: rather than producing progenitors of PAAs, the pharyngeal mesoderm produces ectopic head muscle progenitors. Interestingly, when BMP signal inhibition is relieved, PAA progenitors are capable of reappearing in the pharyngeal mesoderm and develop into growing sprouts. Previous studies have revealed that *npas4l* functions upstream of *etv2*, and *etv2*-deficient vascular progenitors can acquire a skeletal muscle fate, whereas overexpression of *etv2* induces vascular gene expression and converts skeletal muscle cells into functional endothelial cells (Chestnut et al., 2020; Reischauer et al., 2016; Veldman et al., 2013; Yan et al., 2019a). Therefore, when BMP signal is reactivated after the washout of BMP inhibitors, the expression of *npas4l* and its downstream gene *etv2* might be reinduced in the muscle progenitors located in the presumptive PAA clusters and then transdifferentiated into endothelial cells. Additional studies will be required to learn whether BMP-Npas4l-Etv2 pathway is necessary and sufficient to switch the fate of muscle cells into the vascular lineage in the pharyngeal region.

During vertebrate embryonic development, pharyngeal pouches play a central role in organization of the head through expressing signaling molecules such as FGFs and BMPs (Crump et al., 2004; David et al., 2002; Graham, 2008; Holzschuh et al., 2005; Ning et al., 2013). Our data further indicate that pharyngeal pouches induce PAA progenitor specification by expressing BMP ligands. Stem cells or progenitor populations are established in niches where niche factors function to maintain their quiescent state or to induce their proliferation and differentiation for fetal development (Birbrair and Frenette, 2016; Jhala and Vasita, 2015; Scadden, 2006). As endoderm pouches are in close contact with the pharyngeal mesoderm at discrete locations, establishing a physicochemical environment for cell fate determination through the activation of BMP signaling, it is reasonable to hypothesize that pharyngeal pouches provide a niche microenvironment for PAA progenitor specification.

Pouch endoderm expresses several BMP genes including *bmp2a*, *bmp2b*, *bmp4* and *bmp5* (Holzschuh et al., 2005). Using the KalTA4-UAS system to drive pouch-specific expression of miR30-based short hairpin RNAs, we find that both *bmp2a* and *bmp5* are responsible for progenitor specification. This conclusion is in agreement with the fact that *bmp2a^−/−^;bmp5^−/−^* double mutants also exhibit clear defects in PAA formation. Interestingly, the phenotypes seem to be a little more pronounced in the shRNA-expressing embryos. This observation might be due to some unexpected off-target side effects of shRNA-mediated gene silencing. Furthermore, the compensatory expression of other BMP ligands and related components of the BMP signaling pathway in *bmp2a^−/−^;bmp5^−/−^* double mutants may also contribute to this phenomenon. In addition, as most of *bmp2a^−/−^;bmp5^−/−^* double mutants are viable, it will be interesting to investigate whether and how the morphogenesis of the PAAs is recovered in the mutants at later developmental stages in the future.

## MATERIALS AND METHODS

### Ethics statement

Our zebrafish experiments were all approved and carried out in accordance with the Animal Care Committee at the Institute of Zoology, Chinese Academy of Sciences (permission number IOZ-13048).

### Zebrafish lines

Our zebrafish experiments were performed by using the following mutant and transgenic lines: *Tg(nkx2.5:ZsYellow)* (Paffett-Lugassy et al., 2013), *Tg(nkx2.5:kaede)* (Paffett-Lugassy et al., 2013), *Tg(nkx2.3:mCherry)* (Li et al., 2018), *Tg(nkx2.3:KalTA4-p2a-mCherry)* (Li et al., 2018), *Tg(flk:EGFP)*, *Tg(gata1:DsRed)*, *Tg(sox17:GFP)* and *Tg(BRE:EGFP)* (Laux et al., 2011), *Tg(hsp70l:dnBmpr1a-GFP)* (Pyati et al., 2005), *Tg(hsp70l:caBmpr1b-GFP)* (Row and Kimelman, 2009), *Tg(sox10:KalTA4-p2a-mCherry)*, and *Tg(UAS:NTR-mCherry)* and *cloche*^m378^ (Stainier et al., 1995). The *Tg(sox10:KalTA4-p2a-mCherry)* transgenic line was generated by our lab with the *sox10* upstream regulatory sequence as previously described (Carney et al., 2006). The *Tg(UAS:NTR-mCherry)* transgenic line was obtained from China Zebrafish Resource Center. Unless otherwise specified, live embryos were kept at 28.5°C in Holtfreter's solution, and staged based on morphology as previously described (Kimmel et al., 1995).

### Whole-mount *in situ* hybridization

Digoxigenin-UTP-labeled antisense RNA probes for *scl*, *etv2*, *nkx2.5*, *ZsYellow*, *tie1*, *sox17*, *bmp2a*, *bmp2b*, *bmp4, bmp5*,* nkx2.3*,* dlx2a*,* hhex*,* evx1*,* myod1*,* actn3b*,* mef2cb* and *npas4l* were transcribed using MEGAscript Kit (Ambion) according to the manufacturer's instructions. Whole-mount *in situ* hybridization with these RNA probes were performed using the NBT-BCIP substrate.

### Morpholinos and microinjections

Morpholino oligonucleotides (MOs) were purchased from Gene Tools. The standard control MO (5′-CCTCTTACCTCAGTTACAATTTATA-3′) (Dickmeis et al., 2001), *sox32* MO (5′-CAGGGAGCATCCGGTCGAGATACAT-3′) (Dickmeis et al., 2001), *bmp2a* MO (5′-AGTAAACACTTGCTTACCATCATGG-3′) (Naye et al., 2012), *bmp2b* MO (5′-CGCGGACCACGGCGACCATGATC-3′) (Li et al., 2019), *bmp4* MO (5′-GTCTCGACAGAAAATAAAGCATGGG-3′) (Chocron et al., 2007), *bmp5* MO (5′-TTGACCAGGATGATGATGCTTTCAG-3′) (Shih et al., 2017) and *nkx2.5* MO (5′-TGTCAAGGCTCACCTTTTTTCTCTT-3′) (Paffett-Lugassy et al., 2013) were used as previously described. All the MOs were injected at one-cell stage into zebrafish embryos.

### miR30-based shRNAs

The miR30-based shRNAs were designed according to previously published methods (Dow et al., 2012). The target sequences are shown in Table S1. Plasmids expressing shRNAs were microinjected into fertilized eggs at the one-cell stage at the indicated concentrations. The injected embryos were cultured at 28.5°C until needed.

### Generation of *bmp2a* and *bmp5* mutants

We generated *bmp2a* and *bmp5* mutants using the CRISPR/CAS9 technology. We designed the gRNAs of *bmp2a* and *bmp5* using the CRISPR Design website http://crispor.tefor.net/. The Cas9 mRNA and gRNAs were prepared as described previously (Wei et al., 2017), and co-injected into wild-type embryos at the one-cell stage. Embryos were collected to make genomic DNA for genotyping at 24 hpf. For screening of the F1 fish with mutant alleles, genomic DNA was isolated from the tail of individual fish. The forward primer 5′-AAAGACTCGCAATGGCTCG-3′ and reverse primer 5′-TCCCTGTCAGGCATGAAG-3′ were used to amplify *bmp2a* gRNA targeted sequence. And the forward primer 5′-GACTTCTGTGGAGCTGTTTAG-3′ and reverse primer 5′-TGCGTGACCTCTTTACACCAT-3′ were used to amplify *bmp5* gRNA targeted sequence. The amplified fragments were identified with Sanger DNA sequencing for genotyping. F2 embryos were generated by incrossing F1 mutant fishes and genotyped by digesting PCR products with BtsI (NEB, R0667S) and SmaI (NEB, R0141V), respectively.

### Real-time quantitative PCR

Real-time quantitative PCR was performed as previously described (Yan et al., 2019b). The target genes were amplified with the primers listed in Table S2. The expression level of each sample was normalized to β-actin. Statistical analysis was carried out with an unpaired Student's *t*-test.

### Live-embryo imaging and Kaede photoconversion

Live fluorescent embryos were mounted in 1% low-melting agarose in glass-bottomed dish (Solarbio; D35-10-1-N) at indicated stages. *Tg(nkx2.5: ZsYellow)* embryos were imaged and analyzed for the formation of PAAs using a Nikon A1R+ confocal microscope (20× objective). For cell lineage tracing, photoconversion in *Tg(nkx2.5:kaede)* embryos was achieved using a DAPI filter, and the converted embryos were immediately imaged, removed from the agarose and raised in dark conditions until subsequent evaluation. All confocal stack images were processed using Nikon NIS-Elements AR 4.13.00 software.

### Immunofluorescence staining and fluorescent *in situ* hybridization

Immunofluorescence staining was performed as previously described (Ning et al., 2013). Embryos were stained with the following affinity-purified antibodies: anti-GFP (1:1000; A111201, Invitrogen), anti-ZsYellow (1:200; 632475, Clontech), anti-ZsYellow (1:400; TA180004, Origene) and anti-p-Smad1/5/8 (1:200; 9511, Cell Signaling Technology). Fluorescence *in situ* hybridization was performed as previously described (Schoenebeck et al., 2007). Anti-DIG HRP-conjugated Fab fragments (1:400; Roche) were used to detect the digoxigenin (DIG)-labeled probes. Embryos were then incubated with fluorescein (FLU) tyramide (1:100; PerkinElmer) for 3 h at 28.5°C. Next, the embryos were subjected to immunofluorescence after removal of HRP activity.

### Pharmacological treatment and heat shock

For tissue-specific ablation, *Tg(nkx2.3:KalTA4-p2a-mCherry;UAS:NTR-mCherry)* or *Tg(sox10:KalTA4-p2a-mCherry;UAS:NTR-mCherry)* embryos were raised in Holtfreter's solution containing 10 mM MTZ (M1547, Sigma) from bud stage, and then harvested for live imaging or *in situ* hybridization at the indicated stages. To block BMP signaling, embryos were treated with DMH1 (10 μM; D8946, Sigma) or dorsomorphin (10 μM; P5499, Sigma) under dark conditions. *Tg(hsp70l:dnBmpr1a-GFP)* and *Tg(hsp70l:caBmpr1b-GFP)* embryos were subjected to heat shock at 40°C for 20 min at 24 hpf, and then incubated at 28.5°C until harvest.

### Statistical analysis

Statistical analysis was performed with GraphPad Prism software version 5.00 for Macintosh (GraphPad). The numbers of PAA angioblast clusters were counted based on PAA3-PAA6 per embryo. All results are expressed as mean±s.d. Differences between control and treated groups were analyzed with unpaired two-tailed Student's *t*-test. Results were considered statistically significant at *P*<0.05.
